# Molecular and Physiological Responses of *Litopenaeus vannamei* to Nitrogen and Phosphorus Stress

**DOI:** 10.3390/antiox14020194

**Published:** 2025-02-08

**Authors:** Qianqian Zhao, Cun Wei, Jiangling Dou, Yue Sun, Qifan Zeng, Zhenmin Bao

**Affiliations:** 1 MOE Key Laboratory of Marine Genetics and Breeding, College of Marine Life Sciences/Key Laboratory of Tropical Aquatic Germplasm of Hainan Province, Sanya Oceanographic Institution, Ocean University of China, Qingdao, Shandong/Sanya, Hainan 266100/572025, China; zhaoqianqian@stu.ouc.edu.cn (Q.Z.); soiweic@ouc.edu.cn (C.W.);; 2 Southern Marine Science and Engineering Guangdong Laboratory (Guangzhou), Guangzhou 511458, China; 3 Hainan Seed Industry Laboratory, Sanya 572025, China; 4 Hebei Xinhai Aquatic Biotechnology Co., Ltd., Cangzhou 061101, China

**Keywords:** *Litopenaeus vannamei*, nitrogen, phosphorus, physiological, histological, transcriptome

## Abstract

Environmental stressors such as nitrogen and phosphorus play a critical role in regulating the growth and physiological functions of *Litopenaeus vannamei*, a key species in aquaculture. This study investigates the effects of nitrogen and phosphorus stress on shrimp growth, oxidative stress, tissue damage, and molecular mechanisms. Exposure to increasing concentrations of nitrogen and phosphorus significantly reduced growth rates. Oxidative stress markers, including superoxide dismutase (SOD), catalase (CAT), total antioxidant capacity (T-AOC), and malondialdehyde (MDA), indicated heightened oxidative damage under both stress conditions, with nitrogen stress causing more severe responses than phosphorus stress. Histopathological analysis revealed substantial damage to the gills and hepatopancreas, organs essential for respiration and metabolism. Transcriptomic analysis identified differentially expressed genes (DEGs) enriched in apoptosis, lysosome, sphingolipid metabolism, and phagosome pathways, suggesting shared molecular responses to nitrogen and phosphorus stress. The results demonstrate that *L. vannamei* initiates oxidative and immune responses to cope with environmental stressors, but the adaptive capacity remains limited. These findings provide a foundation for understanding the stress tolerance mechanisms in shrimp and inform future strategies for breeding high-resistance strains in aquaculture.

## 1. Introduction

The Pacific white shrimp (*Litopenaeus vannamei*) is one of the most economically significant aquaculture species worldwide, contributing significantly to food security and economic development in coastal regions [1]. Renowned for its adaptability to diverse environmental conditions, rapid growth, and disease resistance, this species accounts for over 50% of global shrimp production [2]. However, intensive aquaculture systems face critical challenges, including high mortality rates due to water quality degradation, disease outbreaks, and stress-induced physiological dysfunction [3]. Among these challenges, excessive nitrogen and phosphorus accumulation—primarily driven by overfeeding and metabolic waste—poses a major threat to shrimp health and farm sustainability [4].

Total nitrogen in aquaculture systems comprises inorganic forms (e.g., ammonia, nitrite, and nitrate) and organic compounds (e.g., urea and proteins) [5]. These nitrogenous substances present significant challenges to the aquaculture of *L. vannamei*, impacting key physiological processes such as development, growth, gas exchange, immune regulation, histological integrity, and metabolism [6,7,8]. Environmental conditions, nitrogen loads, and management practices also influence the concentrations and ratios of these nitrogenous compounds, which significantly interact with the biota, including shrimp [9]. Ammonia, the most toxic nitrogenous waste, disrupts osmoregulation, immune function, and tissue integrity in shrimp [10,11,12]. In aquaculture systems, environmental factors such as low salinity, high water temperature, and elevated pH can exacerbate ammonia toxicity by increasing the NH_3_ proportion. Nitrite impairs oxygen transport by binding to hemocyanin, exacerbating hypoxia [13,14]. While nitrate is less toxic, it can accumulate to harmful levels in poorly managed systems, leading to chronic stress and reduced growth [15]. These nitrogen compounds interact dynamically through microbial nitrification and denitrification [16], creating complex stress scenarios that are rarely studied in combination.

Similarly, phosphorus—essential for energy metabolism (e.g., ATP), skeletal development, oxygen transport, and the maintenance of acid–base equilibrium—becomes problematic when exceeding ecological thresholds [17]. Overfeeding and high-density aquaculture can lead to excessive dissolved phosphorus entering waterbodies to pollute aquatic ecosystems, causing eutrophication and ultimately promoting algal blooms and oxygen depletion [18,19]. Elevated phosphorus levels may also directly impair shrimp physiology, though the mechanisms remain poorly characterized. Together, nitrogen and phosphorus imbalances not only threaten shrimp survival but also escalate production costs and environmental risks in intensive farming [20].

Oxidative stress is a central consequence of nitrogen and phosphorus toxicity. Reactive oxygen species (ROS) generated under these conditions overwhelm antioxidant defenses (e.g., superoxide dismutase, catalase), leading to lipid peroxidation (marked by malondialdehyde, MDA) and DNA damage [21,22,23,24]. Histopathological alterations in vital organs like the hepatopancreas and gills further compromise shrimp resilience, creating a vicious cycle of declining health and productivity [25]. While shrimp exhibit adaptive responses to acute stress, prolonged exposure to sublethal nitrogen and phosphorus levels may exhaust their physiological capacity, highlighting the need for targeted mitigation strategies [7,19].

To address these issues, sustainable aquaculture practices emphasize water quality management, optimized feeding regimes, and breeding for stress tolerance [26]. Innovations such as biofloc technology (BFT)—which leverages microbial communities to recycle nutrients—demonstrate the potential to reduce nitrogen and phosphorus loads while enhancing shrimp growth [27]. However, the molecular and physiological mechanisms underlying shrimp adaptation to these stressors remain underexplored, limiting the development of resilient strains and tailored management protocols. This study investigates the effects of nitrogen and phosphorus stress on *L. vannamei*, focusing on growth performance, oxidative stress biomarkers, histopathological changes, and transcriptomic responses. By integrating physiological and molecular data, we aim to identify threshold concentrations of nitrogen and phosphorus that impair shrimp health and characterize adaptive mechanisms at the antioxidant and gene expression levels. Our findings will advance the understanding of stress tolerance in *L. vannamei* and inform strategies to enhance sustainability across diverse farming systems, from traditional ponds to advanced aquaculture systems.

## 2. Materials and Methods

### 2.1. Experimental Shrimp and Chemical Procedures

All shrimp used in the experiment were obtained from Wenchang, Hainan Province. The shrimp were reared in cement ponds (area 2 m^2^, depth 50 cm), with water pre-treated using mechanical and biological filtration to maintain water quality. Healthy *L. vannamei* (body length 113.90 ± 6.71 mm, weight 9.47 ± 1.05 g) were acclimatized for one week prior to the experiment, during which 50% of the rearing water was exchanged daily. During the experiment, nitrogen and phosphorus concentrations were adjusted using NH_4_Cl, NaNO_2_, NaNO_3_, and K_2_HPO_4_, and their levels were measured with a Hach DR1900 portable spectrophotometer. To maintain water quality, 98% of the rearing water was replaced every 24 h. The system was continuously aerated and water parameters, including salinity (31‰), temperature (26 ± 0.5 °C), and pH (7.7–7.9), were monitored daily.

### 2.2. Experimental Design and Sampling

Based on the safe concentration levels for marine aquaculture, the nitrogen stress experiment was divided into five groups: control, 20 mg/L, 40 mg/L, 60 mg/L, and 80 mg/L. The phosphorus stress experiment was also divided into five groups: control, 10 mg/L, 20 mg/L, 30 mg/L, and 40 mg/L. Each group included three replicates, with 30 shrimp in each replicate. The body length and weight of the shrimps were measured on 0 d and 20 d to assess their growth. At the end of the 20 d exposure period, the hepatopancreas and gills were collected from each shrimp after anesthesia on ice. Part of the dissected hepatopancreas and gills was preserved in 4% paraformaldehyde for tissue sectioning, and the rest of the portion was snap-frozen in liquid nitrogen and stored at −80 °C for further biochemical analysis and RNA extraction. All animal experiments were conducted in accordance with the guidelines and approval of the respective Animal Care and Use Committee of Ocean University of China.

### 2.3. Histological Examination

Histological observations were performed on the hepatopancreas and gills from all groups. The tissues fixed in 4% paraformaldehyde were subjected to a gradient ethanol series (30%, 50%, 70%, 80%, 90%, and 2 h) for dehydration and finally stored in anhydrous ethanol. After clearing with xylene and embedding in paraffin, the samples used for histological observation were cut into slices of 6 μm thickness using a HistoCore AUTOCUT (Leica, Wetzlar, Germany). The sections were then stained with hematoxylin and eosin (H&E), sealed with neutral resin, and observed and photographed by microscope.

### 2.4. Biochemical Analysis

The hepatopancreas and gills were obtained from five nitrogen stress groups and five phosphorus stress groups for biochemical analysis, with three replicates per group and three shrimp per replicate. These tissues were homogenized with 0.9% saline using an electronic homogenizer in an ice water bath and centrifuged at 2500 rpm at 4 °C; then the supernatant was used for antioxidant enzyme activity analysis. The selected biomarkers, including superoxide dismutase (SOD), catalase (CAT), total antioxidant capacity (T-AOC), and malondialdehyde (MDA), are widely recognized indicators of oxidative stress and cellular damage in aquatic organisms. SOD and CAT are key enzymes in the antioxidant defense system, while MDA is a marker of lipid peroxidation, reflecting oxidative damage [28,29]. The activities of SOD, CAT, and T-AOC, as well as the content of MDA in the hepatopancreas and gills, were measured using commercial kits obtained from Nanjing Jiancheng Bioengineering Institute (Nanjing, China). All assays were performed in accordance with the manufacturer’s instructions.

### 2.5. RNA Extraction, Library Construction, and Sequencing Analysis

Total RNA was extracted from the hepatopancreas of the control group and from groups exposed to 80 mg/L nitrogen and 40 mg/L phosphorus, using TRIzol Reagent (Invitrogen, Carlsbad, CA, USA) according to the manufacturer’s protocol. The RNA concentration and purity were assessed by Nanodrop 2000 spectrophotometer (Thermo Fisher Scientific, Waltham, MA, USA). The integrity of the RNA was evaluated using 1.0% agarose gel electrophoresis. High-quality RNA was used for cDNA library construction with the VAHTS Universal V8 RNA-seq Library Prep Kit (Vazyme Biotech Co., Ltd., Nanjing, China), followed by MGI sequencing. The prepared libraries were subsequently sequenced using paired-end 150 bp sequencing (PE150) on the MGI DNBSEQ-T7 platform (BGI Genomics Co., Ltd., Shenzhen, China).

### 2.6. Transcriptome Data Processing and Analysis

Transcriptomic profiling at the mRNA level was prioritized in this study due to its high sensitivity in capturing dynamic transcriptional responses to environmental stress and its cost-effectiveness for large-scale screening of differentially expressed genes (DEGs) [30,31]. While protein-level analyses (e.g., Western blot and ELISA) provide direct functional insights, transcriptomic data remain a robust proxy for identifying key pathways and regulatory networks in non-model organisms like *L. vannamei*, where antibody-based tools are limited. Our approach aligns with recent studies investigating nitrogen and phosphorus stress in crustaceans, which successfully linked mRNA expression patterns to physiological outcomes [13,32]. Future work will integrate proteomic validation to confirm the functional relevance of identified DEGs.

The quality of the raw reads was estimated by fastQC. Adaptors and low-quality reads were trimmed by Trim Galore. Clean reads were aligned with the reference genome of *L. vannamei* using STAR (v2.4.1). Gene expression profiles are presented as transcripts per million (TPM). Differentially expressed genes (DEGs) were identified using DESeq2, with criteria of |log2FoldChange| ≥ 1 and *p* value < 0.05. Volcano plots and clustering heatmaps were generated using R language. Enrichment analysis of Gene Ontology (GO) and Kyoto Encyclopedia of Genes and Genomes (KEGG) was implemented in OmicShare tools (https://www.omicshare.com/tools) [33]. We accessed the tools on 2 September 2024.

### 2.7. qRT-PCR and Statistical Analysis

The total RNA was reverse transcribed into cDNA using a reverse transcription kit (abm, Vancouver, BC, Canada). For each target gene, specific primers were designed using PREMIER 6.0 software, with details provided in Table 1. The qRT-PCR reactions were carried out in triplicate to ensure experimental accuracy. The *β-Actin* gene was used as the internal reference gene for standardizing the expression of 10 target genes. Each experiment was performed with three duplicates. The relative expression of the target genes was calculated using the 2^−ΔΔCt^ comparative Ct technique [34]. In this research, SPSS 22.0 software (IBM, Chicago, IL, USA) was used for all statistical analyses. All data for the tested parameters are presented as mean ± standard deviation (SD). After testing data normality and variance homogeneity, statistical difference was determined by one-way analysis of variance (ANOVA) followed by Duncan’s multiple range test. Significant differences were considered at *p* < 0.05. The data were graphed using Origin 2019 (OriginLab Corp., Northampton, MA, USA).

## 3. Results

### 3.1. Growth Tests and Histopathological Analysis Under Nitrogen and Phosphorus Stress

Growth tests indicate that elevated concentrations of nitrogen and phosphorus in water have a significant inhibitory effect on the growth of shrimp. Analysis of variance (ANOVA) and multiple comparison tests reveal that when the nitrogen concentration exceeds 20 mg/L or the phosphorus concentration exceeds 10 mg/L, the growth rate of shrimp is significantly suppressed (*p* < 0.05). Furthermore, the higher the concentrations of ammonia nitrogen and phosphorus, the more pronounced the inhibitory effect they have on shrimp growth (Table 2).

**Figure 1:**
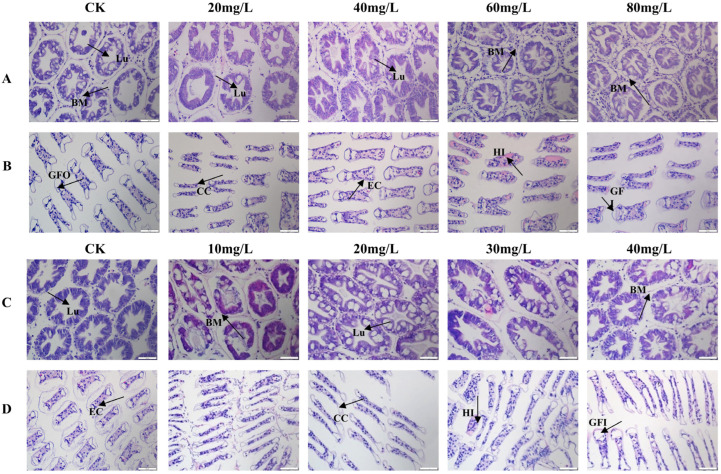
Histological observation of *L. vannamei* under nitrogen and phosphorus stress. (**A**) Hepatopancreas under nitrogen stress. (**B**) Gills under nitrogen stress. (**C**) Hepatopancreas under phosphorus stress. (**D**) Gills under phosphorus stress. Lu: lumen; BM: basement membrane; HI: hemolytic infiltration; CC: Cortex corneum; EC: Epithelial cells; GFI: Gill filament inlet; GFO: Gill filament outlet.

Histopathological analysis was performed on the hepatopancreas and gills. As ammonia nitrogen concentration increased, the hepatic tubules became irregular in shape, with their diameter enlarging at 20 mg/L. At 40 mg/L, the lumens of the tubules began to shrink, accompanied by significant hemocytic infiltration surrounding the tubules. At 80 mg/L, the number of hemocytes within the hepatic tubules markedly increased, with many vacuoles rupturing and some tubules becoming deformed (Figure 1A). In the gills, epithelial cells showed degeneration, resulting in impaired gill inlets and outlets, along with increased infiltrations of hemocytes, in contrast to the control group, where the lamellar structures remained intact and epithelial cells were well-defined. At 60 mg/L of ammonia nitrogen, the gill lamellae were severely damaged, exhibiting extensive infiltrations of hemocytes (Figure 1B).

Under phosphorus stress, the overall structure of the hepatopancreas remained intact; however, the hepatic tubules contracted and deformed as phosphorus concentration increased. At a concentration of 40 mg/L, infiltrating hemocyte densities were observed within the hepatic tubules, along with partial disruption of the basement membranes (Figure 1C). In the gills, the primary structure remained largely intact in the control and low-concentration groups (Figure 1D). However, at a phosphorus concentration of 30 mg/L, the gill lamellae exhibited shrinkage, rupture of epithelial cells, and impaired water exchange, accompanied by internal hemocytic proliferation.

### 3.2. Biochemical Parameters Under Nitrogen and Phosphorus Stress

**Figure 2:**
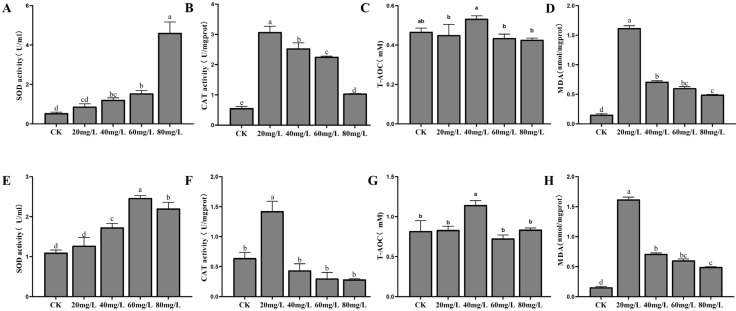
The activities of SOD, CAT, T-AOC, and MDA in the hepatopancreas (**A**–**D**) and gills (**E**–**H**) of *L. vannamei* under nitrogen stress. CK: control. Different letters indicate significant differences (*p*< 0.05).

The antioxidant enzyme activities in the hepatopancreas and gills of *L. vannamei* were measured under nitrogen and phosphorus stress. In the hepatopancreas, SOD activity increased significantly with rising nitrogen concentrations (*p* < 0.05), peaking at 80 mg/L (Figure 2A). CAT activity, while significantly higher than in the control group across all treatments (*p* < 0.05), showed a decreasing trend as nitrogen concentration increased (Figure 2B). T-AOC levels remained stable under all stress conditions (Figure 2C). MDA content followed a pattern of induction, peaking at 20 mg/L, before decreasing. However, MDA levels in all experimental groups were significantly higher than those in the control group (Figure 2D). In the gills, SOD activity initially increased with nitrogen concentration, reaching a peak at 60 mg/L, before declining (Figure 2E). CAT activity peaked at 20 mg/L and then decreased to levels below the control (Figure 2F). T-AOC remained stable, with a significant increase at 40 mg/L compared to other groups (*p* < 0.05) (Figure 2G). MDA content peaked at 20 mg/L before decreasing (Figure 2H).

**Figure 3:**
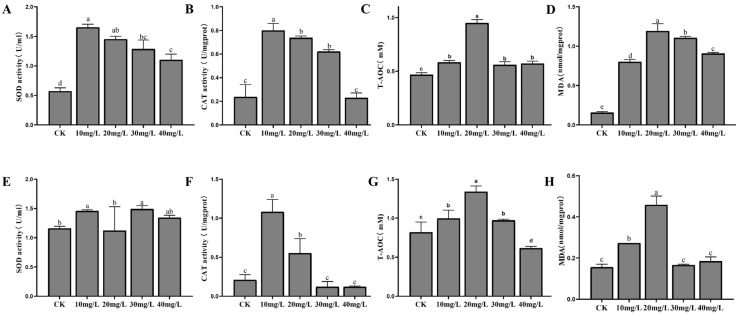
The activities of SOD, CAT, T-AOC, and MDA in the hepatopancreas (**A**–**D**) and gills (**E**–**H**) of *L. vannamei* under phosphorus stress. CK: control. Different letters indicate significant differences (*p* < 0.05).

Antioxidant enzyme responses to phosphorus stress are shown in Figure 3. In the hepatopancreas, SOD activity was significantly higher in all experimental groups compared to the control (*p* < 0.05), although no significant differences were observed among the treatment groups (Figure 3A). CAT activity decreased progressively with increasing phosphorus concentrations, approaching control levels at 80 mg/L (Figure 3B). T-AOC was significantly elevated in the 20 mg/L group compared to both the control and other experimental groups (*p* < 0.05) (Figure 3C). MDA content initially increased, peaking at 20 mg/L, before decreasing, with significantly higher levels observed in all experimental groups relative to the control (*p* < 0.05) (Figure 3D). In the gills, SOD activity fluctuated slightly across the different concentration groups but without significant differences (Figure 3E). CAT activity was significantly higher in the low-concentration groups compared to the control (*p* < 0.05), though no significant differences were detected at higher concentrations (Figure 3F). As phosphorus concentration increased, T-AOC levels in the gills exhibited an initial rise, followed by a decline, with a significant increase observed in the 20 mg/L group (*p* < 0.05) (Figure 3G). MDA content was significantly elevated in the 10 mg/L and 20 mg/L groups compared to the control (*p* < 0.05), but returned to control levels at 30 mg/L and 40 mg/L (Figure 3H).

### 3.3. Transcriptomic Profiling and Identification of DEGs

**Figure 4:**
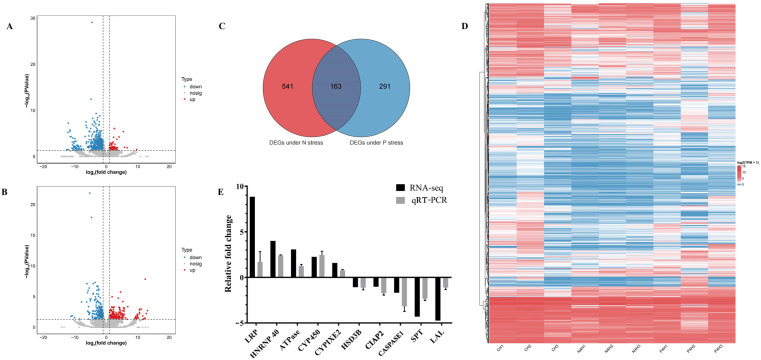
Transcriptomic analysis under nitrogen and phosphorus stress. (**A**,**B**) Volcano plots of DEGs under nitrogen and phosphorus stress. The red points represent significantly upregulated DEGs after exposure, the blue points represent significantly downregulated DEGs, and the gray points indicate non-differentially expressed genes. (**C**) Venn diagram for commonly and exclusively DEGs in two comparison groups. (**D**) Heatmap visualization of DEGs across multiple stress conditions. (**E**) Validation of RNA-seq data using qPCR. CH: control hepatopancreas; N4H: hepatopancreas under 80 mg/L nitrogen stress; P4H: hepatopancreas under 40 mg/L phosphorus stress.

In this study, nine cDNA libraries were constructed, with detailed information provided in Table S1. After filtering out low-quality reads, a total of 546,991,305 clean reads were obtained. High-quality reads accounted for more than 96.90% of the total sequencing reads in each sample. The average Q20 and Q30 values were above 96.35% and 88.88%, respectively, indicating a high level of base-calling accuracy, ensuring that the data were suitable for subsequent analyses. The clean reads were mapped to the *L. vannamei* reference genome, achieving an average mapping rate of 85.43%. Based on the established criteria for differential gene expression screening, 704 DEGs were identified under nitrogen stress, with 92 genes upregulated and 612 downregulated. Under phosphorus stress, 454 DEGs were identified, of which 200 were upregulated and 254 downregulated (Figure 4). To validate the results of RNA-seq, a total of 10 DEGS were randomly selected for qPCR validation, including 5 upregulated genes (*LRP*, *HNRNP40*, *ATPase*, *CYP450*, *CYPIXE2*) and 5 downregulated genes (*HSD3B*, *CIAP2*, *Caspase1*, *SPT*, *LAL*) for quantitative testing. The results demonstrated that the expression trends of these genes were consistent with the changes observed in the RNA-seq data, indicating a high reliability of the RNA-seq results (Figure 4E).

**Figure 5:**
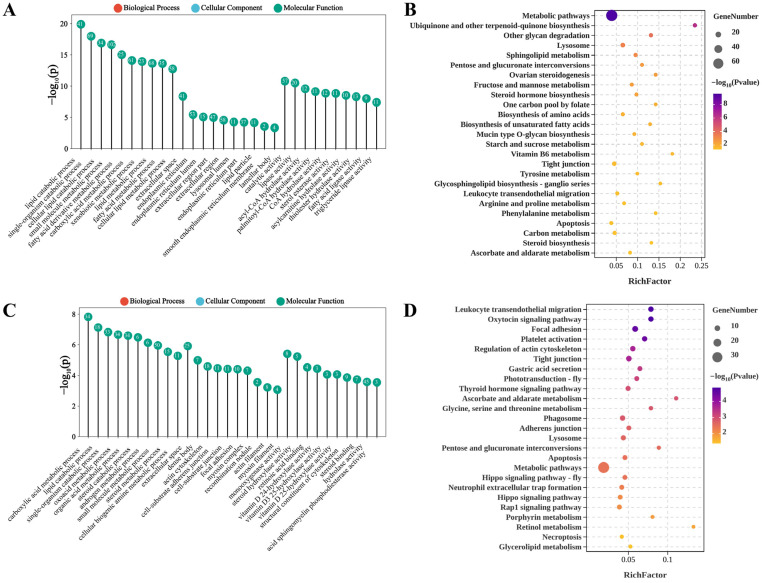
GO and KEGG enrichment analysis of DEGs under nitrogen (**A**,**B**) and phosphorus (**C**,**D**) stress.

Functional enrichment analysis was conducted to explore the molecular events involving DEGs. GO analysis of DEGs under ammonia nitrogen stress highlighted three main categories. Key enriched Biological Processes included lipid catabolic related process. In Cellular Components, enriched terms were extracellular space and endoplasmic reticulum lumen, emphasizing important cellular structures and functions. For Molecular Functions, significant enrichment was found in lipase activity and acyl-CoA hydrolase activity, shedding light on critical metabolic and cellular processes relevant to aquatic environments (Figure 5A). KEGG pathway enrichment analysis revealed that DEGs under nitrogen stress were significantly enriched in 32 pathways (*p* < 0.05). The top enriched pathways included ubiquinone and other terpenoid-quinone biosynthesis, glycan degradation, and sphingolipid metabolism (Figure 5B). These pathways provide insights into the metabolic adaptations and stress responses in shrimp under nitrogen stress.

Under phosphorus stress, GO enrichment analysis revealed several prominent categories. In Biological Processes, the leading terms included carboxylic acid metabolic process and lipid catabolic process. For Cellular Components, enriched terms such as extracellular space, actin cytoskeleton emphasized structural components and interactions within cells. In Molecular Functions, monooxygenase activity, steroid hydroxylase activity, and retinoic acid binding were dominant, reflecting specialized enzymatic activities crucial for physiological regulation in aquatic species (Figure 5C). KEGG pathway analysis indicated that DEGs were significantly enriched in 25 pathways (*p* < 0.05). The top enriched pathways included the oxytocin signaling pathway, focal adhesion, and regulation of actin cytoskeleton (Figure 5D). Notably, pathways including apoptosis, lysosome, glycine, serine and threonine metabolism, metabolic pathways, pentose and glucuronate interconversions, tight junction, ascorbate and aldarate metabolism, focal adhesion, and regulation of actin cytoskeleton were significantly enriched under both stress conditions.

## 4. Discussion

Prolonged exposure to elevated nitrogen and phosphorus levels causes significant physiological and molecular disruptions in *L. vannamei*, with oxidative damage and immune dysfunction emerging as key factors affecting shrimp survival and welfare. In intensive shrimp aquaculture, nitrogen primarily exists in the forms of ammonia (NH_4_^+^/NH_3_), nitrite (NO_2_^−^), and nitrate (NO_3_^−^), with their proportions shifting according to farming system and pond maturity. Traditional pond systems rely on frequent water exchange to manage NH_3_ accumulation [8], whereas biofloc systems (BFT) reduce NH_3_ levels via microbial assimilation but often result in NO_3_^−^ accumulation exceeding 50 mg/L [35]. Recirculating aquaculture systems (RASs) achieve efficient nitrification, maintaining low NH_3_ and NO_2_^−^ levels, but require denitrification strategies to manage NO_3_^−^ accumulation, which can exceed 80% of dissolved inorganic nitrogen [36]. As ponds mature, nitrogen speciation shifts. Early-stage ponds with limited nitrification result in elevated NH_3_ levels, which, combined with high stocking densities, may lead to ammonia toxicity. In mid-stage ponds, NH_3_ is gradually converted to NO_2_^−^, but incomplete nitrification can cause NO_2_^−^ accumulation, necessitating aeration and biofilter optimization. Mature ponds (>60 days) are dominated by NO_3_^−^ (>80% of total nitrogen), but in systems with limited water exchange, NO_3_^−^ accumulation may require denitrification or dilution strategies [37]. Phosphorus, present as inorganic phosphate (P-PO_4_^3−^) and organic P, originates from feed and shrimp metabolism. Excess P-PO_4_^3−^ promotes algal blooms, while organic P accumulates in sediments, influencing long-term nutrient cycling [38]. In traditional pond systems, daily water exchange remains an effective method to mitigate excessive nutrient buildup and ensure sustainable shrimp production [39]. Proper management of nitrogen ratios and phosphorus loads is critical for maintaining water quality and optimizing shrimp growth in intensive systems. The present study demonstrates that prolonged exposure to elevated nitrogen and phosphorus levels induces significant physiological and molecular disruptions in *L. vannamei*, with profound implications for shrimp welfare and survival. While growth suppression under these stressors has been well documented [3,40], our findings emphasize that oxidative damage and immune compromise—rather than mere growth retardation—are the primary drivers of mortality in intensive aquaculture systems.

Oxidative stress occurs when there is an imbalance between oxidants and antioxidants, leading to damage to macromolecules such as DNA, lipids, and proteins, disrupting cellular metabolism and regulation [28]. Under normal conditions, ROS are generated and cleared in a dynamic balance. However, under stress, ROS levels can rise dramatically [41,42]. Studies suggest that low ROS levels enhance immune capabilities in aquatic organisms, but excessive ROS levels can result in lipid peroxidation, damaging cell membranes and causing cellular dysfunction [43]. MDA, a marker of lipid peroxidation, increased in the hepatopancreas and gills across all stress groups, indicating severe lipid peroxidation, a hallmark of irreversible cellular damage [44]. Although MDA levels declined above 20 mg/L, indicating possible adaptation, they remained significantly higher than those of control groups, highlighting the limited adaptive capacity of *L. vannamei* under prolonged nitrogen and phosphorus stress.

This aligns with histopathological observations of hemocytic infiltration, vacuolization, and structural deformation in these organs (Figure 1), which likely impair critical functions such as respiration (gills) and detoxification (hepatopancreas). The gills, critical for respiration, osmoregulation, and ionic balance, showed epithelial cell damage, swelling of gill filaments, hemolytic infiltration, and vacuolization under high nitrogen concentrations. Similar damage has been reported in *L. vannamei* exposed to ammonia, nitrite, and nitrate [15,25]. These impairments likely hinder material exchange and respiratory functions, negatively impacting growth and metabolism. The hepatopancreas, analogous to the vertebrate liver and essential for detoxification, displayed irregularly shaped hepatic tubules, lumen contraction, hemolytic infiltration, and deformation. Such damage is consistent with that observed in *L. vannamei* exposed to heavy metals like zinc and cadmium [45,46], impairing critical physiological and metabolic functions.

In this study, we used several oxidative stress biomarkers, including SOD, CAT, and T-AOC, to assess the effects of nitrogen and phosphorus stress on *L. vannamei*. SOD is a primary defense enzyme that converts superoxide radicals into less harmful substances like hydrogen peroxide and oxygen [29]. In both the hepatopancreas and gills, SOD activity increased with rising nitrogen and phosphorus concentrations, reflecting its key role in managing oxidative stress. In the hepatopancreas, SOD activity under nitrogen stress continued to rise, likely due to its central role in detoxification and metabolism. Under phosphorus stress, SOD activity in the gills showed minor fluctuations, suggesting lower sensitivity to phosphorus. CAT, responsible for converting hydrogen peroxide into water and oxygen, helps reduce oxidative damage [47]. Notably, the decline in CAT activity at high nitrogen concentrations (Figure 2B) suggests a collapse of antioxidant defenses, leaving shrimp vulnerable to cumulative oxidative injury. Such compromised physiological resilience directly correlates with increased mortality rates reported in commercial farms under similar stress conditions [48,49]. T-AOC, which measures the overall antioxidant capacity, initially increased at low stress levels, peaking before stabilizing or declining. This trend suggests that at low stress concentrations, the antioxidant system effectively responds to oxidative stress, but at high concentrations, the system becomes overwhelmed, potentially leading to oxidative damage [50]. The threshold concentrations identified here (nitrogen > 20 mg/L and phosphorus > 10 mg/L) provide actionable benchmarks for water quality monitoring. Beyond growth metrics, our biomarker-based evaluation strategy (SOD, CAT, T-AOC, and MDA) offers a rapid diagnostic tool to assess shrimp welfare and predict mortality risks. For instance, MDA levels exceeding 1.5 nmol/mg protein in the hepatopancreas (Figure 2D,H) could serve as an early warning sign for intervention.

Transcriptomic analysis revealed enrichment of lysosome pathways and apoptosis under both nitrogen and phosphorus stress (Figure 4), implicating dysregulated immune responses. The lysosome pathway is critical for cellular homeostasis, membrane repair, and immune responses. Key genes, such as *ATP6V1* and *GBA*, were upregulated, indicating their crucial roles in phagocytic activity and lipid metabolism, which may enhance stress tolerance [31,51,52,53,54,55]. Conversely, the *SMPD1* gene, which regulates apoptosis and stress responses, was downregulated under both stress conditions [56]. These changes indicate lysosomal dysfunction, which may hinder pathogen clearance by phagocytes. Activation of the apoptosis pathway also reflects systemic cellular stress and that apoptosis—a highly regulated process crucial for development, homeostasis, and immune defense—was significantly enriched under both stress conditions [57,58]. Our findings support that the prolonged exposure to stress led to increased oxidative damage, which in turn resulted in apoptosis [59]. These molecular disruptions align with prior reports linking nitrogenous waste to suppressed lysozyme activity and increased susceptibility to Vibrio infections in *L. vannamei* [12]. Our data suggest that nitrogen stress affects sphingolipid metabolism, a key regulator of cellular function and stress responses, by upregulating key enzymes such as *GBA* and *SPT*, while downregulating *SMPD1*, thereby destabilizing sphingolipid metabolism [60]. This disruption may contribute to the accumulation of harmful intermediates, such as ceramide, which could lead to cellular damage in the hepatopancreas, as observed histologically. Under phosphorus stress, the phagosome pathway, a critical component of the innate immune response, was significantly enriched [13,25]. Environmental stress can suppress immune responses, reducing lysozyme activity and phagocytic function [61,62]. The transcriptomic signatures of stress tolerance (e.g., upregulated *CYPIXE2* and *CYP450*) suggest genetic targets for breeding shrimp strains capable of thriving in innovative management strategies. Integrating such strains with optimized feeding regimes—reducing excess phosphorus inputs—could significantly improve survival rates without compromising productivity [63,64].

In conclusion, this study demonstrates the multifaceted impacts of nitrogen and phosphorus stress on *L. vannamei*, revealing significant physiological, histological, and molecular adaptations. These stressors inhibit growth, elevate oxidative stress, and cause structural damage to the hepatopancreas and gills. Transcriptomic analysis highlights altered gene expression in pathways crucial for apoptosis, lysosome function, and sphingolipid metabolism, suggesting that while cellular mechanisms attempt to cope with stress, their capacity to prevent damage is limited. These findings provide a foundation for breeding *L. vannamei* strains with enhanced resistance to environmental stressors.

## Figures and Tables

**Table 1:** Primers for qRT-PCR.

Gene Accession Number	Primer Names	Primer Sequences (5′-3′)
XM_027375354.1	*CYPIXE2*-Fw	TGGCGATGCTGAAGGAATCTCA
	*CYPIXE2*-Rv	GGCGACCAGGAACAAGACACT
XM_027372322.1	*V-ATPase*-Fw	AAGCTGCCATCCACACTCACAA
	*V-ATPase*-Rv	CCTGGAGCGACCGAGCAATT
XM_027364773.1	*SPT*-Fw	ACGCACACCGACCTGGACTA
	*SPT*-Rv	GAGCGGACTCATCTCGTGGTTG
XM_027354349.1	*HNRNP 40*-Fw	TATGGCGGATACGGTGGCTACG
	*HNRNP 40*-Rv	AGTATGGCTGGTGCCTCGTCTG
XM_027367407.1	*HSD3B*-Fw	AGCACTCTCGCCGTCAAGATG
	*HSD3B*-Rv	GCCATTGTGAGCCTCCAGGA
XM_027383834.1	*Caspase1*-Fw	AGCGTGGTGGTGGTGGTGAT
	*Caspase1*-Rv	GCGGCAGAAGTTGAACAGGAAC
XM_027359957.1	*CYP450*-Fw	TACCGATGCTGCCGCTGATAGG
	*CYP450*-Rv	GCCTGCGAGAACACCTCCTTGA
XM_027359199.1	*C-IAP2*-Fw	CAACGCCGCCAAGAACAACAG
	*C-IAP2*-Rv	ATAACGCTGGTGTCTGCTGGAA
XM_027375397.1	*LRP*-Fw	CCACAGCAGAGGAGGCATTAGT
	*LRP*-Rv	TGGTGAGCAAGGAGAGCATGTT
XM_027357917.1	*LAL*-Fw	TCGGCGGACTTCCAGAGCAT
	*LAL*-Rv	CGTGGTGAACGGTGAGGACATA

**Table 2:** Effects of nitrogen and phosphorus stress on the growth of *L. vannamei*.

	Concentration (mg/L)	Weight Gain (g)	Length Increment (mm)
Nitrogen	Control	7.00 ^a^ ± 1.82	25.79 ^a^ ± 3.57
	20	5.50 ^ab^ ± 1.00	17.78 ^b^ ± 1.40
	40	4.75 ^b^ ± 1.70	13.27 ^b^ ± 6.00
	60	4.25 ^b^ ± 0.50	16.40 ^b^ ± 4.10
	80	3.75 ^b^ ± 0.96	11.90 ^b^ ± 3.52
Phosphorus	Control	7.00 ^a^ ± 1.82	25.80 ^a^ ± 3.58
	10	5.75 ^a^ ± 1.00	16.56 ^b^ ± 3.34
	20	6.50 ^a^ ± 1.70	13.84 ^b^ ± 5.99
	30	4.25 ^ab^ ± 0.95	12.40 ^b^ ± 4.11
	40	3.75 ^b^ ± 1.50	11.89 ^b^ ± 3.52

Note: Different lowercase letters indicate significant differences among stress groups (*p* < 0.05).

## Data Availability

The raw sequencing data generated in this study have been deposited in the NCBI Sequence Read Archive (SRA) under BioProject ID PRJNA1216918.

## Supplementary Materials

The following supporting information can be downloaded at: https://www.mdpi.com/article/10.3390/antiox14020194/s1, Figure S1: Relative expression levels of antioxidant enzyme genes; Table S1: Quantity and quality statistics of sequencing sample reads; Table S2: The detailed information of DEGs; Table S3: Top 50 GO terms in each GO category under nitrogen and phosphorus stress; Table S4: KEGG pathway significantly enriched under nitrogen and phosphorus stress.
