# Supplementary material for: Molecular and Physiological Responses of Litopenaeus vannamei to Nitrogen and Phosphorus Stress
Source: Antioxidants (Basel). 2025 Feb 8;14(2):194. doi: 10.3390/antiox14020194 (PMC11851905; doi:10.3390/antiox14020194)
Supplement: Supplementary file 1 [file antioxidants-14-00194-s001.zip › Table S1.pdf]

Table S1 Quantity and quality statistics of sequencing sample reads

Sample name	Raw Number	Clean Number	Q20（%）	Q30（%）
CH1	20651434	19964984	96.20	88.88
CH2	31394969	30324246	96.35	89.98
CH3	25077334	24118503	97.17	92.14
N4H1	34095520	32822953	96.97	91.35
N4H2	26716727	25857897	97.29	92.21
N4H3	30067706	28993169	97.00	91.36
P4H1	35423752	34557681	96.72	90.55
P4H2	37703017	36267647	97.62	93.01
P4H3	33612039	32803516	96.81	90.87
