# Supplementary figures and images for: Molecular and Physiological Responses of Litopenaeus vannamei to Nitrogen and Phosphorus Stress
Source: Antioxidants (Basel). 2025 Feb 8;14(2):194. doi: 10.3390/antiox14020194 (PMC11851905; doi:10.3390/antiox14020194)

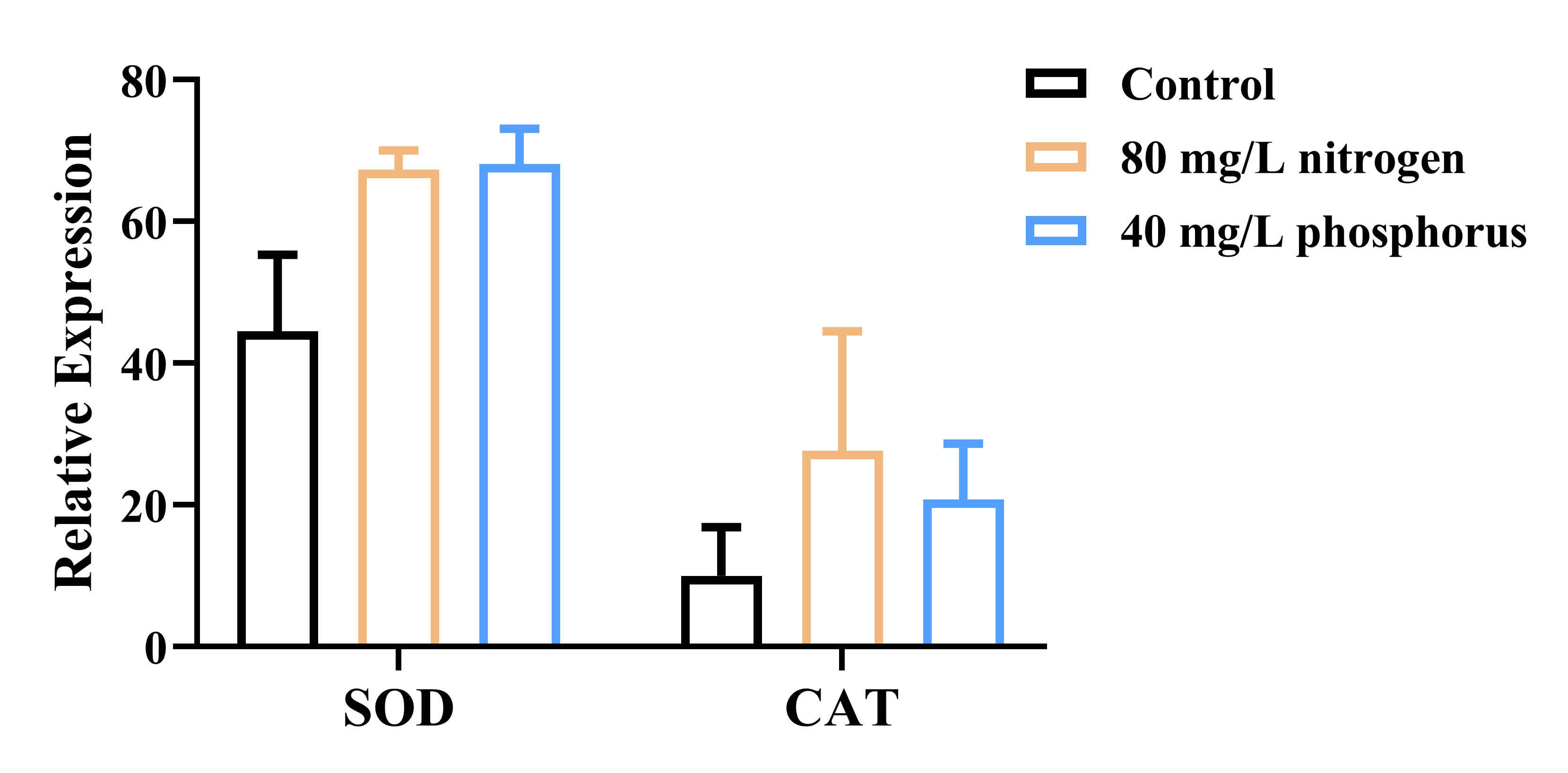

Supplement: Supplementary file 1 [file antioxidants-14-00194-s001.zip › Figure S1.tif]
